# NZ51, a ring-expanded nucleoside analog, inhibits motility and viability of breast cancer cells by targeting the RNA helicase DDX3

**DOI:** 10.18632/oncotarget.4898

**Published:** 2015-08-11

**Authors:** Min Xie, Farhad Vesuna, Mahendran Botlagunta, Guus Martinus Bol, Ashley Irving, Yehudit Bergman, Ramachandra S. Hosmane, Yoshinori Kato, Paul T. Winnard, Venu Raman

**Affiliations:** ^1^ Department of Radiology and Radiological Science, Johns Hopkins University School of Medicine, Baltimore, MD, USA; ^2^ Department of Pathology, University Medical Center Utrecht Cancer Center, GA, Utrecht, The Netherlands; ^3^ Department of Chemistry & Biochemistry, University of Maryland, Baltimore County, MD, USA; ^4^ Department of Oncology, Johns Hopkins University School of Medicine, Baltimore, MD, USA

**Keywords:** ring-expanded nucleoside, RNA helicase DDX3

## Abstract

DDX3X (DDX3), a human RNA helicase, is over expressed in multiple breast cancer cell lines and its expression levels are directly correlated to cellular aggressiveness. NZ51, a ring-expanded nucleoside analogue (REN) has been reported to inhibit the ATP dependent helicase activity of DDX3. Molecular modeling of NZ51 binding to DDX3 indicated that the 5:7-fused imidazodiazepine ring of NZ51 was incorporated into the ATP binding pocket of DDX3. In this study, we investigated the anticancer properties of NZ51 in MCF-7 and MDA-MB-231 breast cancer cell lines. NZ51 treatment decreased cellular motility and cell viability of MCF-7 and MDA-MB-231 cells with IC_50_ values in the low micromolar range. Biological knockdown of DDX3 in MCF-7 and MDA-MB-231 cells resulted in decreased proliferation rates and reduced clonogenicity. In addition, NZ51 was effective in killing breast cancer cells under hypoxic conditions with the same potency as observed during normoxia. Mechanistic studies indicated that NZ51 did not cause DDX3 degradation, but greatly diminished its functionality. Moreover, *in vivo* experiments demonstrated that DDX3 knockdown by shRNA resulted in reduced tumor volume and metastasis without altering tumor vascular volume or permeability-surface area. In initial *in vivo* experiments, NZ51 treatment did not significantly reduce tumor volume. Further studies are needed to optimize drug formulation, dose and delivery. Continuing work will determine the *in vitro*-*in vivo* correlation of NZ51 activity and its utility in a clinical setting.

## INTRODUCTION

Breast cancer is the second leading cause of cancer related death in American women [1]. A number of factors such as nulliparity, age, hormonal factors, alcohol intake and environmental factors, alone or in combination, have been associated with breast cancer incidence and progression [2–4]. Although genetic predispositions to breast cancer are directly correlated to a high likelihood of incidence, these cases represent a relatively low percentage of all breast cancers. Thus, investigations aimed at identifying etiological agents that contribute to breast cancer biogenesis must be undertaken if the vast majority of breast cancer patients are to be helped. One such agent is the environmental pollutant, benzo[a]pyrene diol epoxide (BPDE), which can form stable DNA adducts [5–7]. Previously, our laboratory reported the oncogenic role of BPDE in human breast epithelial cells [8] and identified an RNA helicase, DDX3X (DDX3), as a contributing factor [9]. We demonstrated that DDX3 was upregulated after exposing immortalized non-tumorigenic breast epithelial cells, MCF 10A, to BPDE. Furthermore, stable over-expression of DDX3 in MCF 10A induced an epithelial-to-mesenchymal transition (EMT) and promoted aggressive properties including increased motility, invasiveness and colony formation in soft agar. Also, DDX3 over-expression downregulated E-cadherin expression resulting in the translocation of β-catenin into the nucleus [9]. Importantly, we demonstrated that DDX3 expression directly correlated with high-grade human patient breast tumors indicating a potential role in breast tumorigenesis [9].

DDX3 is a multifunctional protein that belongs to the aspartate-glutamate-alanine-aspartate (D-E-A-D) box RNA helicase family [10, 11]. The putative functions of DDX3 have been associated with a variety of cellular functions, including cell-cycle arrest, cellular proliferation, and apoptosis under various conditions [12–14]. It has also been demonstrated that DDX3 gene mutations are associated with medulloblastoma [15]. Recent evidence indicates that DDX3 interacts with hepatitis C virus (HCV) core protein to maintain both genomic-length HCV RNA and its replicon RNA and that DDX3 is required for HCV virus replication [16, 17]. Evidence also demonstrates that DDX3 is required for the export of unspliced/partially spliced HIV-1 RNAs from the nucleus to the cytoplasm [18] and facilitates translation of HIV-1 mRNAs [19]. It has been reported that knockdown of DDX3 suppressed HIV-1 replication [18, 20]. Based on the findings of DDX3's involvement in HIV, Venkat and colleagues, in pursuit of anti-HIV agents, reported two ring-expanded nucleosides (RENs), one of which is NZ51, as DDX3 inhibitors and demonstrated REN's mode of action as an inhibition of the unwinding activity of DDX3 helicase [21]. RENs mimic the natural 5:6-fused purine nucleoside analogs with a 5:7-fused imidazodiazepine ring system. Recently, a series of tricyclic 5:7:5-fused heterocyclic analogs, which contain the 5:7-fused skeleton, were structurally designed to target DDX3 for anti-cancer activity [22–24]. An additional series of DDX3 inhibitors have been reported, which were found through a high throughput screening study of compound libraries [25–28].

Given the current interest in DDX3 functions in cancer biogenesis, we investigated the utility of NZ51 as a potential anti-cancer drug [21]. NZ51 contains a 5:7-fused imidazodiazepine ring system with a C_18_ alkyl chain tail. We demonstrated that NZ51 treatment decreased DDX3 activity, suppressed cell cycle, inhibited breast cancer cell proliferation, and reduced breast cancer cell motility. However, NZ51 treatment had little effect on primary tumor growth rates in a mouse model system. Investigation is ongoing to determine the possible causes of NZ51 inactivity in animal models.

## RESULTS

### Knockdown of DDX3 decreases cell proliferation and clonogenicity in MCF-7 and MDA-MB-231 cells

**Figure 1:**
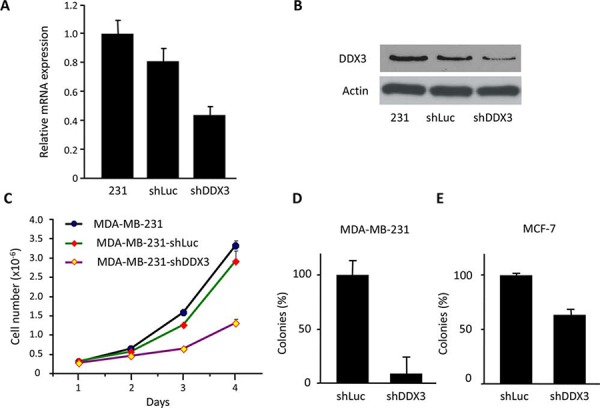
Characterization of MDA-MB-231 DDX3 knockdown cells **A.** qRT-PCR analysis for the expression levels of DDX3 in MDA-MB-231-shDDX3 cell line and the parental MDA-MB-231 cells. Normalization was done by scoring for the expression levels of 36B4, a ribosomal gene. **B.** Immunoblot analysis demonstrating the decreased expression levels of DDX3 protein in MDA-MB-231-shDDX3 (231-shDDX3) cells relative to parental and vector control cells. **C.** Growth curve for MDA-MB-231-shDDX3, parental and vector control cells. **D.** Colony forming assay for MDA-MB-231-shLuc and MDA-MB-231-shDDX3 cells. **E.** Colony forming assay for MCF-7-shLuc and MCF-7-shDDX3 cells.

To evaluate the function of DDX3 expression in cancer cell proliferation, DDX3 was knocked down in MCF-7 and MDA-MB-231 breast cancer cell lines with a lentiviral shDDX3 construct. As shown in Figure 1A–1B, MDA-MB-231 had relatively high levels of endogenous DDX3 and the corresponding shDDX3 treatment knocked down DDX3 in MDA-MB-231 cells to an estimated 50–60% at both the mRNA and protein level. A subsequent study indicated that the growth rate of MDA-MB-231-shDDX3 cells was slower, compared to control (Figure 1C). In order to check the effect of DDX3 knockdown on clonogenic ability, MDA-MB-231-shDDX3 and MCF-7-shDDX3 cell lines along with control cell lines were cultured for 1–2 weeks. We observed that DDX3 knockdown caused over 90% decrease in colony forming ability for MDA-MB-231 cells and a 40% decrease for MCF-7 cells (Figure 1D–1E). These results are an indication that DDX3 has important functions during breast cancer cell growth and proliferation.

### Knockdown of DDX3 in MDA-MB-231 cells decreases metastasis in a preclinical breast cancer model

**Figure 2:**
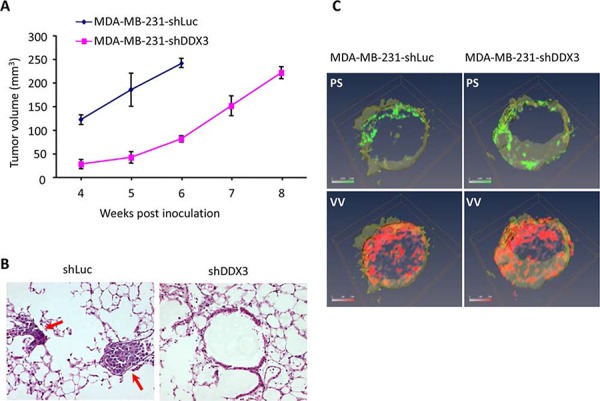
The effect of DDX3 knockdown of MDA-MB-231 on tumor growth, metastatic potential and tumor microenvironment **A.** Graphical depiction of primary tumor volumes of the respective xenografts over an eight-week period. **B.** Post mortem H&E staining analysis of lungs from orthotopic primary tumor xenografts generated with either MDA-MB-231-shLuc or MDA-MB-231-shDDX3 cells. Red arrow points to the foci of the tumor cells in the lungs (*p* = 0.0377). **C.** Images of tumor vascular volume (*VV*) and permeability-surface area products (*PS*) obtained by MRI for control and MDA-MB-231shDDX3 cells. Red and green channels represent the distribution of *VV* and *PS*, respectively.

To biologically characterize the role of DDX3 in breast cancer biogenesis, we generated xenografts in female SCID mice using MDA-MB-231-shDDX3 or MDA-MB-231-shLuc cells. As shown in Figure 2A, the tumor growth rate for MDA-MB-231-shDDX3 (60% decrease in DDX3 transcript), as anticipated from the *in vitro* study (Figure 1D), was initially slower than that of MDA-MB-231-shLuc during the first 6 weeks of the study. After 6 weeks of growth, the growth rate of the MDA-MB-231-shDDX3 derived tumors was similar to MDA-MB-231-shLuc derived tumors. To validate the potential differential metastatic properties of these tumors (Figure 2B), animals were euthanized when tumor volumes reached approximately 250 mm^3^. Autopsies revealed that animals inoculated with MDA-MB-231-shLuc cells had, on average, 17 metastatic foci in the lungs while mice that received MDA-MB-231-shDDX3 cells (Figure 2B) had fewer metastatic lesions. This indicates that lowering DDX3 expression abrogates metastatic progression under these conditions.

### DDX3 knockdown effect on tumor vascular permeability and vascular volume

As tumors generated from DDX3 knockdown cells exhibited reduced metastatic load, we wanted to determine whether there were any alterations to the tumor microenvironment. To characterize these changes, a magnetic resonance imaging (MRI) study was carried out to visualize tumor vasculature, vascular volume (*VV*) and permeability-surface area products (*PS*) (Figure 2C). A comparison of the permeability-surface area products and vascular volume of tumors generated using MDA-MB-231 and MDA-MB-231-shDDX3 cells (Figure 2C), indicated no significant differences in these parameters in our model. This indicated that DDX3 expression may not be the primary driver of vascularization in these tumors and the reduced metastatic load could be due to altered cellular changes induced by decreased DDX3 expression.

### Molecular modeling of interactions of NZ51 with DDX3

**Figure 3:**
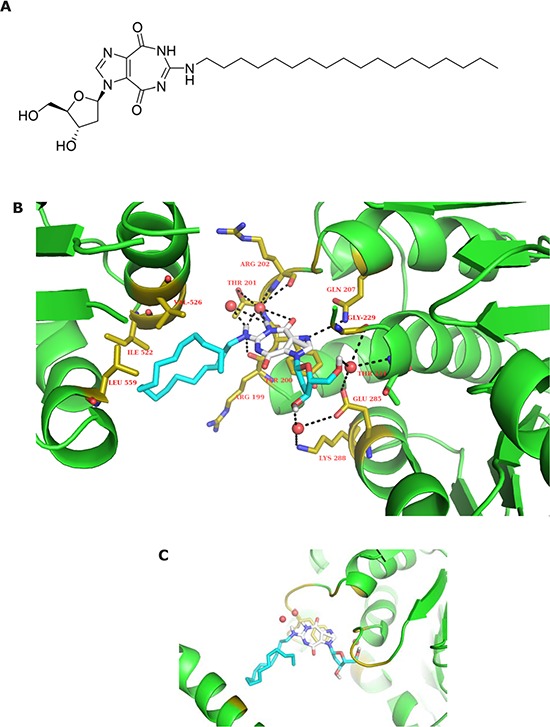
Structure of NZ51 and molecular modeling structure of NZ51 binding to DDX3 **A.** Chemical structure of NZ51. **B.** The energy-minimized complex of NZ51 and DDX3, indicating that NZ51 fits into ATP binding pocket. **C.** The aerial view of imidazole-diazepine ring system stacked with phenol ring of Tyr 200. Generation of a model of the interaction of drug with DDX3 is based on the reported crystal structure of DDX3 (PDB ID: 2I4I).

To understand the inhibition activity of NZ51 on DDX3, as demonstrated in a previous study [21], using the Autodock Vina molecular modeling program, we generated an energy-minimized NZ51-DDX3 interaction model with binding energy of −6 to −6.5 Kcal/mol using an open crystal structure of DDX3 (PDB ID: 2I4I) [29]. The docking results indicated that NZ51 (Figure 3A) fits into the ATP binding site with its lipophilic octadecyl tail (blue) close to lipophilic amino acid residues including Ile 522, Val 526 and Leu 559 (within 4 angstrom) (Figure 3B). The hydrogen binding interactions between NZ51 main skeleton and polar amino acid residues in the binding pocket include interactions of HOH 685 with both N-H at position 6 of NZ51 (2.4 Å) and N-H at position 7 of NZ51 (3.2 Å), HOH 683 with N-H at position 6 (2.2 Å), N-H at position 7 (2.0 Å), Thr 201-O-H (3.9 Å) as well as Arg 202 backbone C=O (3.3 Å), Arg 202 backbone C=O and N-H at position 7 (4.0 Å), Arg 199 backbone C=O and N-H at position 7 (3.9 Å), Thr 204 N-H and O=C at position 8 (3.9 Å), Gln 207 side chain N-H_2_ and N-3 (imidazole) of NZ51 (3.4 Å), HOH 782 with O-3′ (ribose) of NZ51 (2.7Å), Lys 288 N-H3^+^ (4.2 Å) and Glu 285 side chain C=O (4.0 Å), HOH 609 with O-5′ (ribose) of NZ51 (2.8 Å), Gly 229 C=O (3.5 Å) and Glu 285 side chain C=O (3.7 Å). Also, the aerial view indicates that the imidazole-diazepine ring system can be stacked with phenol ring of Tyr 200 to enhance tighter binding (Figure 3C). In conclusion, the modeling study indicates that thermodynamically favorable, hydrophobic and hydrophilic interactions between NZ51 and the amino acid residues of the ATP binding site of DDX3 can occur and thus, such a favorable binding of NZ51 at this site may explain the inhibition of ATP-dependent helicase activity of DDX3.

### NZ51 treatment inhibits growth of aggressive breast cancer cells

**Figure 4:**
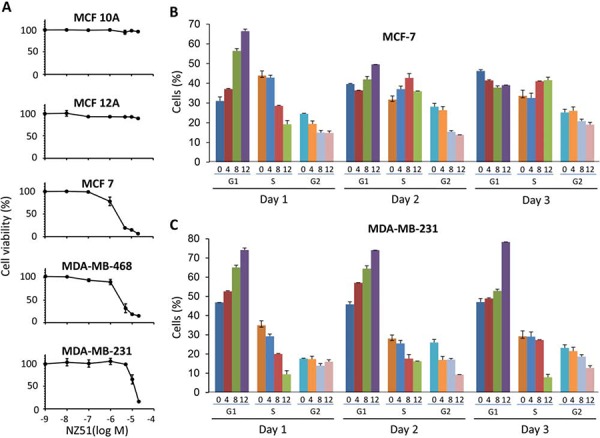
Effect of NZ51 on cell viability of normal breast cell lines and breast cancer cell lines as well as on cell cycle progression of MCF-7 and MDA-MB-231 **A.** Cell viability assay of normal immortalized breast cell lines and breast cancer cell lines incubated with different concentrations of NZ51 for 72 h at 0.001, 0.01, 0.1, 1, 5, 10 and 20 μM. **B.** Flow cytometry analysis determinations of the percentage of MCF-7 cancer cells in G1, G2/M, and S phase of the cell cycle during three consecutive days of treatment with NZ51 at 0, 4, 8, 12 μM. The latter concentrations are indicated under the bars of each graph. **C.** Flow cytometry analysis performed with MDA-MB-231 cancer cells.

To study the effects of NZ51 on cell growth and proliferation, non-tumorigenic immortalized breast cells (MCF 10A and MCF 12A) and breast cancer cells (MCF-7, MDA-MB-468, and MDA-MB-231) were used. Previously, our laboratory demonstrated that DDX3 protein expression levels directly correlates to the tumorigenicity and aggressiveness of breast cancer cell lines [9]. To investigate the cell growth inhibitory activity of NZ51 on these various breast cancer cell lines, cell viability assays were performed following treatment with increasing concentrations of NZ51. The results showed that the viabilities of non-tumorigenic immortalized cell lines with very low DDX3 expression, MCF 10A and MCF 12A, were unaffected at all NZ51 concentrations tested (Figure 4A). However, NZ51 inhibited cell proliferation of MCF-7, MDA-MB-468 and MDA-MB-231 cells with IC_50_ values of 2 μM, 3 μM, and 10 μM respectively. These results indicated that the possible inhibition of DDX3 activity by NZ51 in breast cancer cells resulted in growth inhibition.

### NZ51 treatment causes cell arrest at G1 phase

To determine whether NZ51's inhibition was related to arrest of the cell cycle, flow cytometry was used to quantitate the cell cycle phase percentage distributions following treatment with different concentrations of NZ51. As shown in Figure 4B, at day 1, treating MCF-7 cells with increasing concentrations of NZ51 resulted in an increase in the percentage of cells in G1 phase with proportional decreases in the percentage of cells in S and G2/M phases. By day 2, increasing NZ51 concentration had less of an effect on the percentage of MCF-7 cells in G1 or S phases but did lower the percentage of cells entering G2/M phase (Figure 4B). Day 3 mirrored the results seen at day 2 for MCF-7 cells but the degree of change in cells entering G2/M was much smaller. Thus, NZ51 treatment of MCF-7 cells had its most effective impact on the cell cycle during day 1 where 8 and 12 μM concentrations of NZ51 caused an accumulation of cells in G1 arrest and a loss of cells in S phases. For MDA-MB-231 cells, treatment with 12 μM NZ51 had the largest impact by day 3 at which time most of the cells were arrested in G1 phase and the least were in S and G2/M phases (Figure 4C). In general, these results indicated that treatment of these breast cancer cell lines with 12 μM of NZ51 had a differential effect on the cell cycle after day 1. The comparatively more aggressive MDA-MB-231 cell line was more profoundly affected at later time points. This might be a reflection of its relatively higher expression of DDX3 in MDA-MB-231 cells compared to MCF-7 cells.

### NZ51 diminishes “wound-healing” capabilities MCF-7 and MDA-MB-231 cell lines

**Figure 5:**
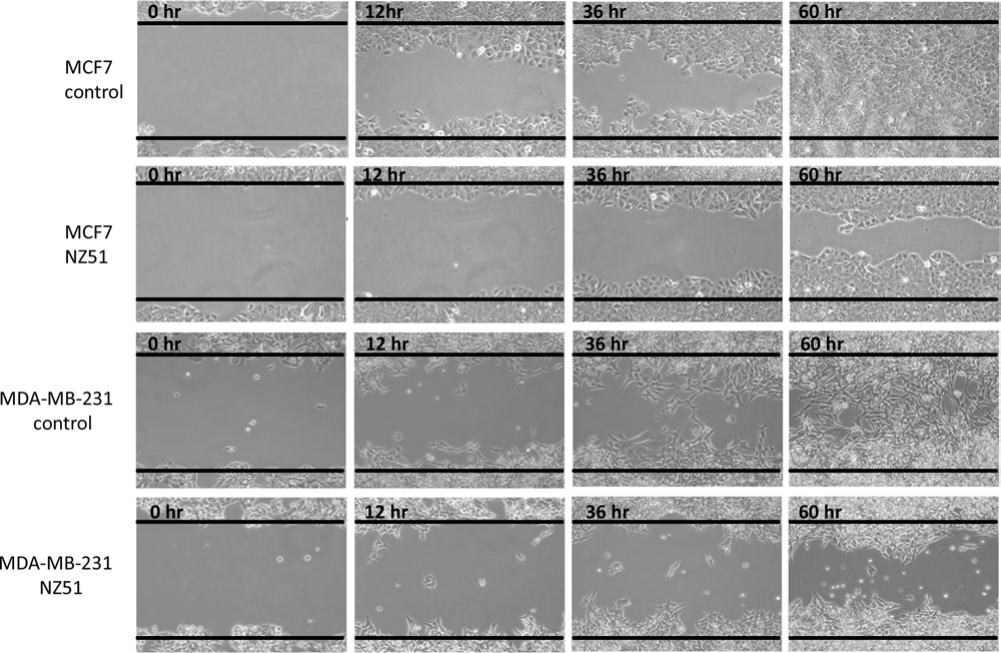
*In vitro* wound-healing/scratch assay **A.** MCF-7 cancer cells. **B.** MCF-7 cancer cells treated with 10 μM of NZ51. **C.** MDA-MB-231 cancer cells. **D.** MDA-MB-231 cancer cells treated with 10 μM of NZ51. Photomicrographs were obtained at the indicated time points using a 10X objective on a Nikon eclipse TS100 inverted microscope and recorded using NIS-Elements F 3.2 software.

To further examine how NZ51 treatment inhibited cell proliferation along with cell motility, “wound-healing” assays were performed. The results indicated that DMSO treated MCF-7 cells nearly completely filled the “wound” by 60 hr (Figure 5). In stark contrast, treatment of MCF-7 cells with NZ51 greatly hindered the motility/proliferation of these cells and 20–30% of the wound remained unfilled at 60 h (Figure 5). Similar results were observed in the NZ51 treated MDA-MB-231 cells versus DMSO treated cells (Figure 5) and again motility/proliferation was hindered as can be seen at the 60 h time point where 40–50% of the “wound” remains unresolved (Figure 5). These results are a further indication that NZ51 treatment of breast cancer cell lines impairs their ability to proliferate as well as hindering their motility.

### Cellular effects of NZ51 on breast cancer cell lines

**Figure 6:**
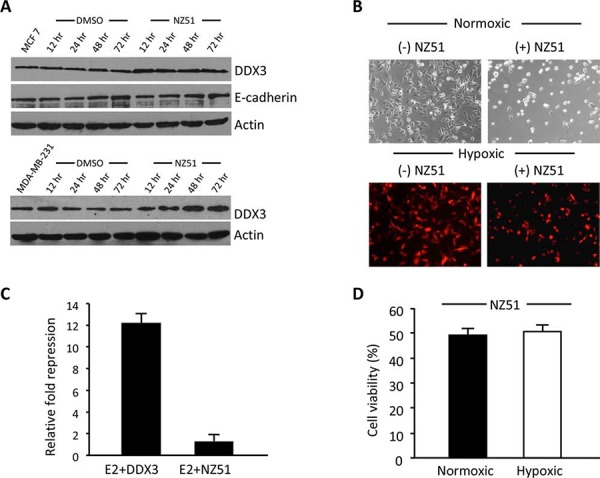
NZ51 stabilizes DDX3 with inactivation of its function and is not affected by hypoxia **A.** Immunoblot of MCF-7 and MDA-MB-231 total protein extracts from control and NZ51 treated scored for DDX3 and E-cadherin expression levels at the indicated post treatment times with β-actin as loading control. **B.** Photomicrographs of MDA-MB-231 cells before and after 72 h incubation with NZ51 under normoxic and hypoxic conditions. **C.** MCF-7 cells were either co-transfected with an E-cadherin promoter reporter construct (E2) along with a CMV-DDX3 expression vector or with only E2, followed by incubation with NZ51. Luciferase activity was estimated 24 h following NZ51 addition. The fold repression was calculated against the luciferase activity of E2 construct alone in MCF-7 cells. **D.** MTS assay results following NZ51 incubation under normoxic and hypoxic conditions for 72 hr.

As NZ51 showed robust growth inhibition of breast cancer cell lines, we investigated the possible mechanism of action of NZ51. Based on the molecular modeling profile, NZ51 binds the nucleoside-binding site of DDX3. This could lead to either the destabilization/degradation of DDX3 or abrogation of its functional activity. Towards determining the action of NZ51, MCF-7 and MDA-MB-231 cells were incubated with 5 μM and 10 μM of NZ51 respectively for different time intervals (12, 24, 48 and 72 hr). Following incubation, total proteins were extracted and scored on immunoblots for DDX3 levels. As shown in Figure 6A, DDX3 levels were higher in treated cells (as early as 12 hr) than the DMSO controls. This appears to indicate that the binding of NZ51 to DDX3 results in a decreased turnover of DDX3 protein. As reported earlier, over expression of DDX3 in MCF 10A cells decreased expression of E-cadherin levels [9]. To confirm if the resulting DDX3-NZ51 complex was functionally active, we scored for E-cadherin levels, a down-stream target of DDX3 [9]. As demonstrated in Figure 6A, E-cadherin levels remained constant, indicating the DDX3-NZ51 complex was not functionally active. In addition, functional E-cadherin promoter-reporter assays supported our initial findings that the elevated DDX3 levels in the NZ51 treated MCF-7 cells were not functionally active (Figure 6C). Taken together, these results indicate that binding of NZ51 to DDX3, although reducing DDX3 degradation, makes the complex functionally inactive in breast cancer cell lines.

### Effect of hypoxia on the functional activity of NZ51 to induce cell death

During solid tumor biogenesis, regions of hypoxia develop within the tumor due to inadequate and poorly formed vasculature. These regions have been shown to be resistant to chemotherapy and radiation and have also been closely linked to malignant progression [30, 31]. To evaluate the efficacy of NZ51 to induce cell death under hypoxic conditions, MDA-MB-231 cells engineered to express a hypoxia induced fluorescent protein (tdTomato) were used (Figure 6B) [32]. Following confirmation of hypoxic conditions by bright red fluorescence, NZ51 was added to the cells at 10 μM and incubated for 72 hr. As shown in Figure 6B & 6D, NZ51 was able to decrease the viable cell fraction by approximately 50%, indicating that NZ51 retains its activity under both normoxic and hypoxic conditions, thus making it an excellent drug candidate for breast cancer treatment.

### *In vivo* study of NZ51 effect on tumor volume of MDA-MB-231 cells

As NZ51 exhibited excellent cytotoxic effects against MCF-7 and MDA-MB-231 cells *in vitro*, we investigated whether NZ51 treatment would reduce tumor volume in mouse models. Weekly measurements of the tumor volumes after injection of DMSO and NZ51 into MDA-MB-231 inoculated athymic nu/nu mice were performed (Supplementary Figure 1). However there was no significant difference in tumor growth rates between control and NZ51 treated mice. This could be due to several factors including lack of efficient drug delivery, rapid clearance, and drug degradation.

## DISCUSSION

Recently, our laboratory reported that DDX3 could have important cellular functions that participate in or even drive breast cancer carcinogenesis [9]. Our data also demonstrated that DDX3 expression levels can be directly correlated with the invasiveness of patient breast cancer samples [9]. We also demonstrated that stable over-expression of DDX3 in MCF 10A cells induced an EMT and promoted aggressive properties such as increased motility and invasion as well as the ability to form colonies in soft agar, all of which are characteristic features of cellular transformation.

The present work provides corroborating evidence that DDX3 is required for cancer cell proliferation both *in vitro* and *in vivo* and targeting DDX3 with a small molecule inhibitor, NZ51, could be a viable option for breast cancer treatment. As a first step towards determining the biological functions of DDX3, we knocked down DDX3 mRNA and protein levels in both MCF-7 and MDA-MB-231 cell lines. The obtained data showed that the proliferation rate of MDA-MB-231-shDDX3 cells was significantly reduced. Additionally, knockdown of DDX3 decreased the clonogenicity of both MCF-7 and MDA-MB-231 cells. Moreover, an orthotopic xenograft mouse model was used to assess the effect of DDX3 knockdown on the *in vivo* growth rate and metastatic potential of MDA-MB-231-shDDX3 versus MDA-MB-231 tumors. This study was further supported by the clonogenicity data indicating that DDX3 expression levels could be an important marker that is associated with tumor growth rate. Most importantly, the decreased DDX3 protein expression levels of MDA-MB-231-shDDX3 tumors resulted in reduced metastatic lesions to the lungs indicating a possible function of DDX3 during metastatic progression. Interestingly, decreased DDX3 expression levels in MDA-MB-231-shDDX3 cells did not change the tumor vascular volume or the permeability-surface area product indicating that there is little or no role of DDX3 in promoting angiogenesis during tumor growth and progression in the model system used [33, 34].

A small molecule inhibitor of DDX3, referred to as NZ51, was initially investigated as an anti-HIV-1 agent targeting DDX3. The rationale for this targeted approach was based on the finding that DDX3 was necessary for the transporting of unspliced or partially spliced HIV-1 virus mRNA from the nucleus to cytoplasm and thus vital to HIV-1's replication cycle [18]. Our molecular modeling studies of a DDX3 interaction with NZ51 showed that NZ51 can be incorporated into the DDX3 ATP binding region with multiple favorable hydrogen and hydrophobic bonding interactions which supported the hypothesis that NZ51 can directly interact with DDX3 and inhibit its activity by blocking or displacing ATP and thus arrogate DDX3 unwinding helicase function.

Given our initial finding that DDX3 participates in cellular transformation, proliferation, motility, tumor growth rate, and metastatic progression, we explored NZ51 as a potential inhibitor of DDX3. We found that NZ51 inhibited cell growth of breast cancer cell lines with high DDX3 expression (MCF-7, MDA-MB-468 and MDA-MB-231) but not against two non-cancerous breast cell lines (MCF 10A and MCF 12A) with low DDX3 expression. We further evaluated the effects of NZ51 on the cell cycle, which indicated that NZ51 mediated an increase in the percentage of MCF-7 and MDA-MB-231cells in G1 phase and the accumulation was directly correlated to the NZ51concentration. However, for MCF-7 cells this phenomenon was only confirmed at day 1 of NZ51 treatment and there were no significant changes at day 2 or day 3 after treatment. This could be due to less effective drug concentration caused by NZ51 metabolism and/or sequestration into a subcellular compartment. In further support for the utility of NZ51, a wound-healing assay demonstrated that NZ51 decreased the cell migration of MCF-7 and MDA-MB-231 cells (Figure 5). At the cellular level, NZ51 may be stabilizing DDX3 protein levels, i.e., slow its turnover rate, while abolishing its function as determined by an E-cadherin reporter assays. This could be due to altered protein confirmation in the presence of NZ51 that abrogates DDX3's normal interactions with cellular macromolecules, thereby blocking its degradation and inactivating its functions. Under these same conditions no change in E-cadherin or actin levels was seen, which points to the fact that NZ51 is not likely a general inhibitor of cellular protein degradation processes.

As is customary with any chemotherapeutic agent, it is advantageous to demonstrate if the drug will be active in a tumor's hypoxic pockets since hypoxic regions are well known to contribute to radiation and chemotherapy resistance as well as metastatic progression. Thus, drugs that are effective in killing hypoxic tumor cells greatly aid in eradicating the cancer. Our results demonstrated that NZ51 is active in both normoxic and hypoxic environments, which indicate that NZ51 can kill breast cancer cells regardless of the oxygenation status of the cells.

As the data from the present studies indicated that NZ51 could be a very promising candidate for anticancer drug development, we investigated the antitumor activity *in vivo* to confirm whether NZ51 can reduce the tumor volume in a mouse model. However, our *in vivo* mouse model drug therapy experiment showed that NZ51 treated mice developed tumors with sizes comparable to those of untreated animals. Considering the chemical structure of NZ51, it is possible that the water insolubility may hinder its distribution *in vivo* and development of a drug delivery system such as PLGA nanoparticles may overcome this problem.

In conclusion, the evidence from this study indicates that NZ51 shows excellent *in vitro* inhibition of proliferation rates of cancer cells expressing relatively high levels of DDX3. However, further experiments will have to be carried out to determine its efficacy in animals. Structure function correlations need to be elucidated and potential modification to NZ51 carried out to determine whether an increased efficacy can be reached, which would facilitate its translation into a clinical setting.

## MATERIALS AND METHODS

### Cell culture and reagents

MCF-7 cells were maintained in modified Eagle's medium (MEM) containing 10% fetal bovine serum (FBS). MDA-MB-231 cells were maintained in Roswell Park Memorial Institute medium (RPMI-1640) containing 10% FBS. MCF 10A and MCF 12A cells were cultured in a 1:1 mixture of Dulbecco's modified Eagle's medium and F12 medium (DMEM/F12 50/50) supplemented with 5% horse serum, hydrocortisone (0.5 μg/ml), insulin (10 μg/ml), epidermal growth factor (20 ng/ml), penicillin (100 I.U./ml), and streptomycin (100 μg/ml). MDA-MB-468 cells were cultured in Leibovitz's L-15 medium with 10% FBS. All medium are from Cellgro, Herndon, VA, USA. All cell lines were obtained from ATCC and cultured under standard sterile cell culture conditions in a humidified incubator at 37°C and 5% CO_2_.

### Cell proliferation assay

MCF 10A, MCF 12A, MCF-7, MDA-MB-468 and MDA-MB-231 cells were cultured at a density of 1 × 10^3^ cells per well in flat-bottomed 96-well plates. After 72 h of treatment with NZ51 at 0.001, 0.01, 0.1, 1, 5, 10 and 20 μM, MTS solution was added to each well, cells were incubated for an additional 2 h and the cell viability was determined by measuring the absorbance at 490 nm using a Victor3 V1420 multiplate counter (Perkin Elmer, Waltham, MA). MDA-MB-231 cells (parental, control and shDDX3) were plated at 1 × 10^5^ cells per well in 6-well plates and allowed to grow for 4 days. MTS assays were performed after 1, 2, 3 and 4 days of growth.

### Flow cytometry analysis

MCF-7 (5 × 10^4^ cells per well) and MDA-MB-231 cells (1 × 10^5^ cells per well) were plated in 6-well plates and treated with NZ51 at concentrations of 0, 4, 8 and 12 μM. Cell cycle analysis were carried out as previously described [35]. Briefly, cells were trypsinized after 24, 48, and 72 h of growth and fixed in 70% ethanol overnight at −20°C. Fixed cells were washed with PBS and resuspended in DNA staining solution (5 μg/ml propidium iodide, 0.5 mg/ml RNase A) for 1 h at room temperature. Cell cycle acquisition was performed on a FACScan I or FACSCalibur instrument (BD Biosciences, San Jose, CA). Independent triplicate experiments were performed. Data was analyzed using FlowJo software (Tree Star Inc., Ashland, OR).

### Colony-formation assays

MCF-7, MCF-7-shDDX3, MDA-MB-231, and MDA-MB231-shDDX3 cells were seeded at a density of 200 cells/well in 6 well plates in 2 mL of medium. Following incubation for 2 weeks, the colonies were stained with 0.5% crystal violet and counted.

### Wound-healing assay

MCF-7 and MDA-MB-231 cells were grown in 6-well plates until about 70–80% confluency was reached at which point a 10 μL pipette tip was used to create a scratch/wound with clear edges across the width of a well. Wells were treated either with vehicle control (DMSO) or 10 μM NZ51 and photomicrographs were taken over a 60 h time period. A Nikon TS100 inverted microscope was used to measure and photograph the cell migration from the wound/scratch edge every 12 hr. All experiments were performed in triplicates.

### DDX3 knockdown by shRNA

DDX3 shRNA lentiviral constructs have been described previously [9]. Briefly, the construct has a U6 promoter driving DDX3 shRNA and a PGK promoter driving EGFP. The control was a shLuc lentiviral vector. Stable shLuc and shDDX3 clones were generated in MCF-7 (MCF-7-shDDX3 and MCF-7-shLuc) and MDA-MB-231 (MDA-MB-231-shDDX3 and MDA-MB-231-shLuc) cell lines using lentivirus transduction.

### Immunoblotting

Following treatment of NZ51, cells were washed with PBS, trypsinized and harvested for protein expression analysis. Standard SDS-PAGE and immunoblotting protocols were followed throughout. The primary antibodies used were monoclonal antibodies against DDX3, actin and E-cadherin. The secondary antibody used was anti-mouse-HRP antibody and visualized using Chemiluminescence (Pierce, Rockford, IL).

### Molecular modeling

The Autodock Vina program was used to perform the molecular modeling. The crystal structure of DDX3 (in the open confirmation) was obtained from the Protein Data Bank (PDB ID: 2I4I) [29]. NZ51, was docked into the nucleoside-binding site. The grid box of the ATP-binding site was generated according to the position of AMP within its confirmed binding site. Autodock uses a scoring function based on amber force field, which estimates the lowest free energy of binding of a ligand (NZ51) to its target (DDX3).

### Quantitative reverse transcription polymerase chain reaction

RNA from breast cancer cells was extracted according to the manufacturer's instructions (Qiagen, Valencia, CA) and cDNA was generated with qScript cDNA synthesis kit (Quanta BioSciences, Gaithersburg, MD), followed by qPCR using SYBR green (Quanta BioSciences, Gaithersburg, MD) on an iCycler5 (Bio-Rad, Hercules, CA). Amplification of 36B4, a housekeeping gene, was used for normalizing gene expression values.

### Orthotopic xenograft models

All animal experiments were conducted in accordance with guidelines from the Johns Hopkins Animal Care and Use Committee; mice were maintained under pathogen-free conditions and given food and water ad libitum.

### Evaluation of DDX3-knockdown on metastatic progression

One million of MDA-MB-231-shLuc and MDA-MB-231 shDDX3 cells were inoculated in the second left thoracic mammary fat pad of 4–6 weeks old female SCID mice. Mice were monitored weekly and tumor volumes were measured and evaluated by formula: V = (ab^2^)/2 [36]. Lung metastases were stained using H&E staining of lung sections.

### Measurements of tumor vascular parameters

Human breast cancer MDA-MB-231 and MDA-MB-231 shDDX3 cells were inoculated in the left upper thoracic mammary fat pad of 4–6 week old female SCID mice. To visualize tumor vasculature, vascular volume (*VV*) and permeability-surface area product (*PS*), magnetic resonance imaging (MRI) was carried out on a horizontal 30-cm bore 9.4T Bruker Biospec preclinical scanner pre- and post-intravenous administration of an albumin-GdDTPA macromolecular contrast agent into mice. The imaging procedure for *VV* and *PS* measurements was detailed in our earlier reports [33, 34], except that we used 250, 500, and 1,000 ms for relaxation delays. Three-dimensional angiograms were acquired using a gradient-echo sequence with the following parameters: echo time = 2.5 ms, repetition time = 8 ms, number of averages = 4, and matrix size = 256 × 128 × 64. Data were analyzed with in-house software written in the IDL programming environment (Exelis Visual Information Solution, Inc., Boulder, CO) and 3D images were visualized with the Amira graphic package (Visage Imaging Inc., San Diego, CA).

### NZ51 treatment efficacy

Athymic NCr-nu/nu mice 4–5 weeks old were purchased from NCI Fredrick. The mice were anesthetized using 2.5% isoflurane and one million MDA-MB-231 cells were injected in the second thoracic fat pad using an insulin syringe. Mice were randomly grouped into two groups. Treatment started 18 days after tumor cell inoculation with daily DMSO (vehicle control) or NZ51 (80 mg/kg) intraperitoneal injections for 18 days. Tumor dimensions were measured weekly with a digital caliper and volumes calculated using the formula: V = (ab^2^)/2 [36]. Mice were sacrificed when the longest tumor diameter reached 10 mm.

### Statistical analysis

All experiments were performed a minimum of three times. Linear regression analysis was used to compute the concentration of test agent needed to reduce mitochondrial activity by 50%, termed the midpoint cytotoxicity. Unpaired, *t*-test was performed for flow cytometric analysis. *P*-values ≤ 0.05% were considered significant. *VV* and *PS* values were compared between MDA-MB-231 and MDA-MB-231-shDDX3 xenografts using nonparametric Mann-Whitney *U* test.

## SUPPLEMENTARY FIGURE


