# Supplementary material for: NZ51, a ring-expanded nucleoside analog, inhibits motility and viability of breast cancer cells by targeting the RNA helicase DDX3
Source: Oncotarget. 2015 Aug 11;6(30):29901–13. doi: 10.18632/oncotarget.4898 (PMC4745771; doi:10.18632/oncotarget.4898)
Supplement: Supplementary file 1 [file oncotarget-06-29901-s001.pdf]

SUPPLEMENTARY FIGURE

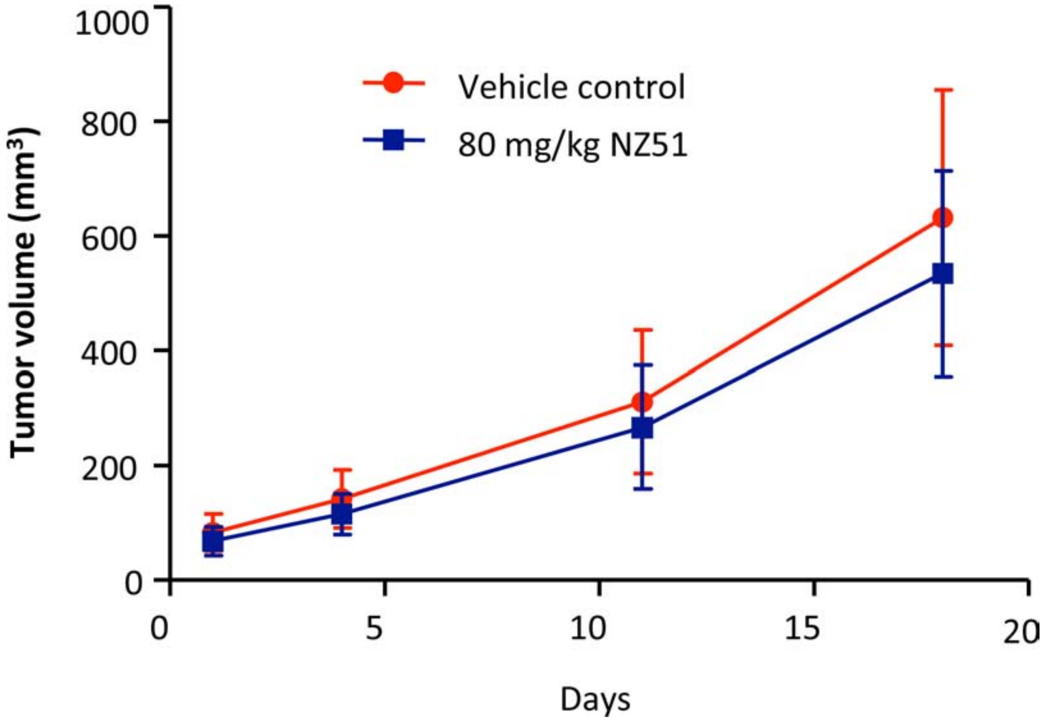

Supplementary Figure S1: *In vivo* study of NZ51 effect on tumor growth rate. Tumor volumes of female athymic nu/nu mice (two groups of 5 mice) inoculated with MDA-MB-231 cells and treated with NZ51 (80 mg/kg) or vehicle when tumor volumes had reached ~75 mm^3.
